# Deletion of Rac1GTPase in the Myeloid Lineage Protects against Inflammation-Mediated Kidney Injury in Mice

**DOI:** 10.1371/journal.pone.0150886

**Published:** 2016-03-03

**Authors:** Miki Nagase, Hidetake Kurihara, Atsu Aiba, Morag J. Young, Tatsuo Sakai

**Affiliations:** 1 Department of Anatomy and Life Structure, Juntendo University School of Medicine, Tokyo, Japan; 2 Laboratory of Animal Resources, Center for Disease Biology and Integrative Medicine, Graduate School of Medicine, The University of Tokyo, Tokyo, Japan; 3 Centre for Endocrinology and Metabolism, Hudson Institute of Medical Research and Monash University, Clayton, Victoria, Australia; University of Kentucky, UNITED STATES

## Abstract

Macrophage-mediated inflammation has been implicated in various kidney diseases. We previously reported that Rac1, a Rho family small GTP-binding protein, was overactivated in several chronic kidney disease models, and that Rac1 inhibitors ameliorated renal injury, in part via inhibition of inflammation, but the detailed mechanisms have not been clarified. In the present study, we examined whether Rac1 in macrophages effects cytokine production and the inflammatory mechanisms contributing to kidney derangement. Myeloid-selective Rac1 flox control (M-Rac1 FC) and knockout (M-Rac1 KO) mice were generated using the cre-loxP system. Renal function under basal conditions did not differ between M-Rac1 FC and KO mice. Accordingly, lipopolysaccharide (LPS)-evoked kidney injury model was created. LPS elevated blood urea nitrogen and serum creatinine, enhanced expressions of kidney injury biomarkers, *Kim-1* and *Ngal*, and promoted tubular injury in M-Rac1 FC mice. By contrast, deletion of myeloid Rac1 almost completely prevented the LPS-mediated renal impairment. LPS triggered a marked induction of macrophage-derived inflammatory cytokines, *IL-6* and *TNFα*, in M-Rac1 FC mice, which was accompanied by Rac1 activation, stimulation of reduced nicotinamide-adenine dinucleotide phosphate (NADPH) oxidase, and reactive oxygen species overproduction. These changes were inhibited in M-Rac1 KO mice. LPS evoked F4/80-positive macrophages accumulation in the kidney, which was not affected by myeloid Rac1 deficiency. We further tested the role of Rac1 signaling in cytokine production using macrophage cell line, RAW264.7. Exposure to LPS increased *IL-6* and *TNFα* mRNA expression. The LPS-driven cytokine induction was dose-dependently blocked by the Rac1 inhibitor EHT1864, NADPH oxidase inhibitor diphenyleneiodonium, and NF-κB inhibitor BAY11-7082. In conclusion, genetic ablation of Rac1 in the myeloid lineage protected against LPS-induced renal inflammation and injury, by suppressing macrophage-derived cytokines, *IL-6* and *TNFα*, without blocking recruitment. Our data suggest that Rac1 in macrophage is a novel target for the treatment of kidney disease through inhibition of cytokine production.

## Introduction

Macrophage infiltration is a hallmark of kidney disease.[1] A number of renal insults stimulate macrophage recruitment to the kidney, including immune complexes, microorganisms, high glucose levels, renin-angiotensin-aldosterone system, excessive salt intake, ischemia, oxidative stress, and proteinuria.[2–4] Activated macrophages release cytokines and chemokines, and accelerate renal inflammation, fibrosis, and damage. In addition to these pathogenic, classically activated M1 macrophages, there is an alternatively activated M2 phenotype, which participates in the resolution of inflammation, tissue repair, and remodeling.[5, 6] Recent studies also highlighted the role of F4/80^bright^ tissue macrophages originated from yolk sac-derived erythro-myeloid progenitors.[7] Strategies for targeting pathogenic M1 macrophages [8, 9] or macrophage-derived M1 cytokines and chemokine receptors,[10, 11] or for utilizing reparative M2 macrophages [12, 13] are beneficial for the treatment of renal inflammation and damage.

Rac1, a member of the Rho-family of small GTPases, is a multi-functional molecule that regulates actin cytoskeletal dynamics,[14] generates reactive oxygen species (ROS),[15] and affects many cellular processes, including transcription, cell migration, adhesion, and proliferation.[16] The *in vivo* significance of Rac1 was previously substantiated using gene targeted mice. Systemic Rac1 knockout (KO) mice were embryonic lethal due to germ-layer formation defects.[17] Subsequently, several conditional KO mice were generated. Deletion of Rac1 in the epidermis leads to epidermal stem cell loss and defects in hair follicles.[18] Dorsal telencephalon-restricted Rac1 KO mice manifest agenesis of commissural axons due to failure of midline crossing during cortical development.[19] Vascular endothelial-specific Rac1 ablation results in embryonic lethality around embryonic day E9.5 with defective vascular development,[20] while hemizygous excision of Rac1 in the endothelium protects against brain injury after focal cerebral ischemia.[21] Cardiomyocyte-specific Rac1 deficient mice were resistant to cardiac hypertrophy.[22] These findings indicate a variety of cell-specific (patho)physiological roles of Rac1.

In the kidney, Rac1 is shown to act as a regulatory subunit of reduced nicotinamide-adenine dinucleotide phosphate (NADPH) oxidase, and the resultant ROS exert proinflammatory responses via NF-κB activation.[23, 24] We previously reported that Rac1 inhibitors conferred renoprotection, in part by suppressing inflammatory signals in several chronic kidney disease models.[25–28]. However, the detailed mechanisms of Rac1-evoked inflammation in kidney diseases, including cellular target, have not been clarified. In the present study, we focused on Rac1 in macrophages, and investigated its role in lipopolysaccharide (LPS)-mediated inflammation and kidney injury using conditional KO mice.

## Materials and Methods

### Ethics Statement

This study was performed in strict accordance with the guidelines of the Animal Research Committee of Juntendo University, and protocols were approved by the Animal Research Committee of Juntendo University (Permit Number: Animal 270019, DNA 26–11, 26–18). Animals were sacrificed under sodium pentobarbital anesthesia, and all efforts were made to minimize suffering.

### Antibodies

We used the following antibodies; rat anti-mouse F4/80 monoclonal antibody (Cl:A3-1, AbD Serotec, MCA497); rat anti-mouse Ly-6B.2 monoclonal antibody (7/4, AbD Serotec, MCA771); mouse anti-human Rac1 monoclonal antibody (23A8, Merck Millipore, 05–389); mouse anti-β actin monoclonal antibody (AC-15, Sigma, A5441); goat anti-p47phox polyclonal antibody (Sigma, SAB2500674); rabbit anti-human NF-κB p65 monoclonal antibody (D14E12, Cell Signaling Technology, 8242); rabbit anti-human AIF monoclonal antibody (D39D2, Cell Signaling Technology, 5318); rabbit anti-human histone H3 monoclonal antibody (D1H2, Cell Signaling Technology, 4499); donkey anti-Rabbit IgG, HRP-Linked F(ab’)_2_ Fragment (GE Healthcare, NA9340); sheep anti-Mouse IgG, HRP-Linked F(ab’)_2_ Fragment (GE Healthcare, NA9310), and donkey anti-goat IgG-HRP (Santa Cruz Biotechnology, sc-2020).

### Targeted inactivation of Rac1 in the myeloid lineage in mice

Rac1-floxed (Rac1^flox/flox^) mice containing loxP sites flanking the entire exon 1 of the *Rac1* gene (originally a hybrid of C57BL/6, 129Ola, and ICR, which was backcrossed 5 generations with C57BL/6) [19] were crossed with transgenic mice harboring the cre recombinase under the control of the myeloid lineage-specific promoter, lysozyme M (LysM^cre/+^) (C57BL/6J background),[29, 30] generating myeloid-specific Rac1 KO (LysM^cre/+^ Rac1^flox/flox^: M-Rac1 KO) mice. LysM^+/+^ Rac1^flox/flox^ littermates were used as flox control (M-Rac1 FC). Genotype was determined by PCR using tail genomic DNA and the following primers: cre (Fwd: 5′-AGGTTCGTTCACTCATGGA-3′, Rev: 5′-TCGACCAGTTTAGTTACCC-3′) and Rac1 (Fwd: 5′-ATTTTCTAGATTCCACTTGTGAAC-3′, Rev: 5′-ATCCCTACTTCCTTCCAACTC-3′). Animals were housed in a room maintained at constant temperature, humidity, and light cycle (12-h light/dark).

### LPS-induced kidney injury model

LPS from Escherichia coli 0111:B4 (Sigma) was dissolved in phosphate buffered saline (PBS) at a concentration of 0.25 mg/ml. Male M-Rac1 FC and KO littermates (16 to 24 weeks old) were given a single intraperitoneal injection of LPS (5 mg/kg) or PBS (Vehicle). To avoid dehydration, PBS was injected at 24 h. At 48 h after LPS injection, blood was collected. Kidneys were harvested after perfusion with 50 ml of saline. Blood urea nitrogen (BUN) and serum creatinine were measured at SRL laboratory (Tokyo, Japan).

### Cell culture

Mouse monocyte/macrophage cell line, RAW264.7 (TIB-71, ATCC), were cultured in RPMI1640 medium (Life Technologies) supplemented with 10% fetal bovine serum (FBS: HyClone) and antibiotics. Cells were stimulated with LPS (1 to 1000 ng/ml) for the indicated time. In some experiments, cells were pretreated with the Rac1 inhibitor EHT1864 (Tocris), the NF-κB inhibitor BAY11-7082 (Wako Pure Chemical Industries), or the NADPH oxidase inhibitor diphenyleneiodonium (DPI: Sigma) for 2 h, then subjected to LPS. EHT1864 associates with Rac1 tightly and causes the release of bound GTP, thus prevents effector interaction.[31] BAY11-7082 is an irreversible inhibitor of IκB kinase α and phosphorylation of cytokine-inducible IκBα, thus inactivates NF-κB. DPI, an inhibitor of flavoenzymes, blocks NADPH oxidase activity.

### Preparation of bone marrow-derived macrophages (BMDM)

L929 cells (CCL-1) were purchased from ATCC. L929-cell conditioned medium (LCCM) was obtained as a source of granulocyte/macrophage colony stimulating factor, by cultivating L929 cells in RPMI supplemented with 10% FBS and antibiotics for 4 days and additional 3 days. Bone marrow cells were collected in a sterile condition from the femur and tibia of M-Rac1 FC and KO mice, and cultured in RPMI containing 30% LCCM, 20% FBS, and antibiotics for 7 days.[30] Purified BMDM were cultured in RPMI 1640 containing 5% LCCM, 10% FBS, and antibiotics, and RNA and protein were extracted on the next day.

### Gene expression analysis

Total RNA was extracted using an RNeasy mini kit (Qiagen), and reverse-transcribed to cDNA with High Capacity cDNA Reverse Transcription Kit (Applied Biosystems). Gene expression was determined by real-time quantitative RT-PCR using 7500 Fast Real-Time PCR System (Applied Biosystems).[27] We basically used TaqMan chemistry and commercially available primers and probe sets. For *cre* expression, we used SYBR Green chemistry and the following primers (Fwd: 5’- GCGTTCGAACGCACTGATTTC -3’, Rev: 5’- TACACCAGAGACGGAAATCCA -3’).

### Histological analysis

Kidneys were fixed in 4% paraformaldehyde solution. For morphologic evaluations, we stained paraffin sections (4 μm) with hematoxylin-eosin. The degree of tubular injury was semiquantitatively assessed using at least 10 randomly selected fields in each specimen in a blinded manner. Tubular injury was graded (0 to 4) on the basis of the percentage of tubular dilation, atrophy, cellularity, basement membrane thickening, or sloughing, as follows: 0, intact; 1, <10%; 2, 10–25%; 3, 25–50%; 4, 50–75%; 5, >75% of the tubules were affected.[27]

### Immunohistochemistry

Paraformaldehyde-fixed cryosections (5 μm thick) were treated with 3% hydrogen peroxide, avidin-biotin blocking solution, 0.5% blocking reagent (Sigma), then incubated with rat anti-mouse F4/80 (1:200) or rat anti-mouse Ly-6B.2 (7/4, 1:200), and subsequently with biotin-labeled secondary antibodies.[28, 32] Immunosignals were detected using a VECTASTAIN Elite ABC kit (Vector Laboratories) and a metal-enhanced DAB kit (Thermo Fisher Scientific), counterstained with hematoxylin, and observed under light microscope (Olympus BX51). For quantitative evaluation of macrophage and neutrophil accumulation, the numbers of F4/80-positive and Ly-6B.2-positive cells were counted in 10 randomly selected high-power fields (× 200). [28]

### Western blotting

Western blotting was performed as described previously.[25] Whole cell lysates were extracted using MLB buffer containing 25 mM HEPES (pH7.5), 150 mM NaCl, 1% NP-40, 10% glycerol, 10 mM MgCl_2_, and Protease Inhibitor cocktail. Nuclear protein was obtained using Nuclear/Cytosol Fractionation Kit (BioVision). For membrane fractionation, samples were homogenized in M1 buffer that contained 20 mM Tris (pH 7.4), 2 mM MgCl_2_, 250 mM sucrose, and Protease Inhibitor cocktail. Nuclei and unlysed cells were removed by low-speed centrifugation (500 x g), and the supernatant was centrifuged at 100,000 x g. The pellet was resuspended in M2 buffer that contained 40 mM Tris (pH 7.9), 260 mM sucrose, 1% Triton X-100, and Protease Inhibitor cocktail.[33] The nitrocellulose membrane was immunoblotted with mouse anti-Rac1 (1:2000),[27] mouse anti-β actin (1:2000), goat anti-p47phox (1:2000, http://www.sigmaaldrich.com/catalog/product/sigma/sab2500674?lang=en&region=US), rabbit anti-NF-kB p65 (1:1000, http://media.cellsignal.com/pdf/8242.pdf), rabbit anti-AIF (1:1000, http://media.cellsignal.com/pdf/5318.pdf), or rabbit anti-human histone H3 (1:1000, http://media.cellsignal.com/pdf/4499.pdf). After incubation with HRP-linked anti-rabbit IgG (1:4000), anti-mouse IgG (1:4000), or anti-goat IgG (1:5000),[27] signals were visualized with ECL, ECL Prime, or ECL Advance Western blotting detection system GE Healthcare).

### Rac1 activation assay

Rac1 activity was evaluated by GST pull-down assay.[25] Briefly, lysates were incubated with agarose beads coupled with GST fusion protein corresponding to the p21-binding domain of PAK1 (Merck Millipore). The precipitated proteins were subjected to SDS-PAGE and immunoblotting.

### Evaluation of NADPH oxidase activity

NADPH oxidase activity was measured using bis-N-methylacridinium nitrate (lucigenin) chemiluminescence.[33] Briefly, the kidney was homogenized in HBSS buffer containing 10 mM HEPES (pH 7.4) (50 mg tissue weight/ml buffer). After incubation for 20 min at 37°C, NADPH (100 μM) and lucigenin (5 μM) were added to the sample. Immediately, the chemiluminescence was recorded for every 30 sec at an interval of 30 sec for 5 min with a luminometer (MiniLumat LB9506; Berthold Technologies). NADPH oxidase activity was expressed as chemiluminescence of superoxide (RLU: relative light unit) standardized by tissue weight. We confirmed that the oxidative burst was completely inhibited by a specific NADPH oxidase inhibitor DPI (100 μM).

### Thiobarbituric acid reactive substances (TBARS) assay

ROS in the kidney were evaluated as malonyldialdehyde (MDA) using TBARS Assay Kit (Zeptometrix). Briefly, the kidney homogenate (50 mg/ml PBS) was mixed with sodium dodecyl sulfate and thiobarbituric acid-buffer reagent, and incubated at 95°C for 60 min. The sample was cooled to room temperature, and centrifuged at 3,000 rpm for 15 min. The absorbance of the supernatant was measured at 532 nm in a spectrophotometer (NanoDrop 2000, Thermo Scientific). The concentration of TBARS (μM) was calculated from a standard curve of MDA.

### Statistical analysis

Values are expressed as means ± s.e.m. For multiple comparisons, statistical analysis was performed by two-way ANOVA or one-way ANOVA followed by Bonferroni's *post hoc* test. Student’s unpaired *t*-test was used for comparison between the two groups. Histological data were analyzed using nonparametric analysis with Kruskal-Wallis test, followed by Mann-Whitney U test. *P* Values < 0.05 were considered to be significant.

## Results

### Establishment of myeloid-selective Rac1 KO mice

**Fig 1:**
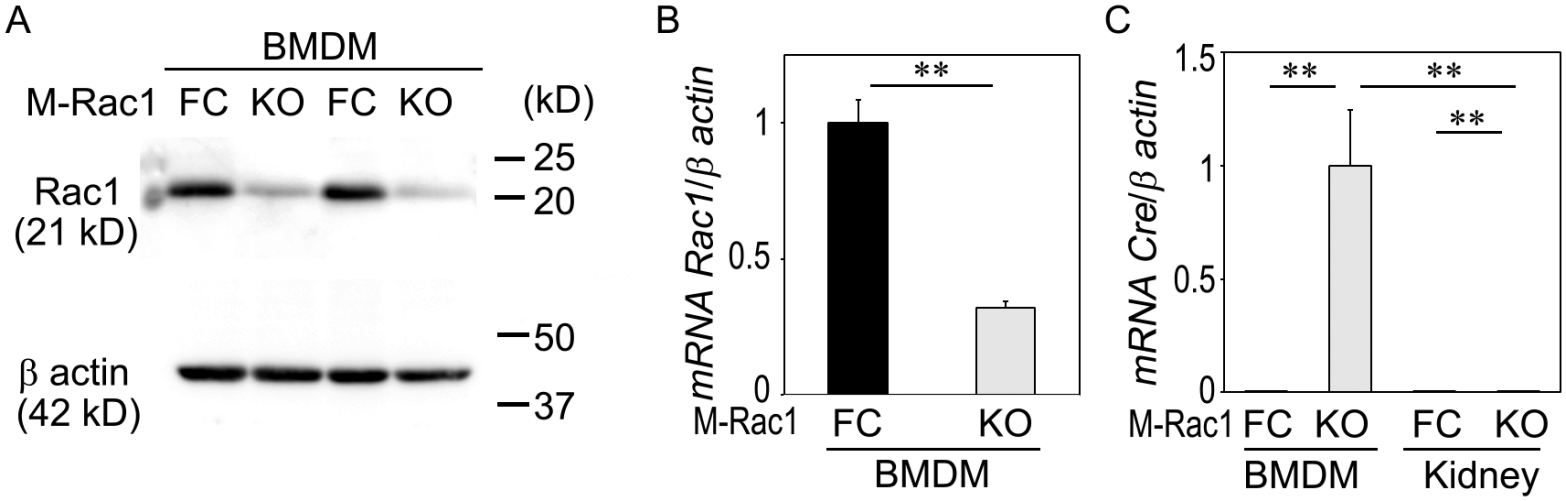
Validation of myeloid-specific Rac1 knockout mice. (A and B) Immunoblotting (A) and qPCR (B) analyses of Rac1 in the bone marrow-derived macrophages (BMDM) from myeloid-selective Rac1 knockout (M-Rac1 KO) and flox control (M-Rac1 FC) mice. β actin was used as a loading control. Data are means ± s.e.m. Statistical analysis was performed using unpaired *t*-test. **P < 0.01. *n* = 3 per each group. (C) Expression of *cre* mRNA in the BMDM and kidneys from M-Rac1 FC and KO mice. Statistical analysis was performed by two-way ANOVA, *P* < 0.01 genotype effect, *P* < 0.01 organ effect, *P* < 0.01 interaction effect. **P < 0.01 by Bonferroni's *post hoc* test. *n* = 3 per each group.

M-Rac1 FC and KO mice were born in the Mendelian ratio. Immunoblotting and qPCR analyses revealed reduced expressions of Rac1 and abundant and selective induction of *cre* in BMDM, validating cell type-specific Rac1 depletion in the myeloid cells (Fig 1A–1C). *Cre* transcripts were not detected in any organs in M-Rac1 FC mice. On the other hand, *cre* mRNA was present in the kidney of M-Rac1 KO mice, although the expression level was much lower (0.0023 ± 0.0003 fold compared to BMDM), reflecting the presence of a small number of cre-positive tissue macrophages in the kidney.

### Myeloid Rac1 deletion protects against LPS-induced kidney injury

Under basal conditions, renal function (BUN, serum creatinine, Na, K, Cl concentrations, creatinine clearance, urinary albumin excretion) and histology did not significantly differ between M-Rac1 FC and KO mice (S1 Table). Accordingly, the mice were subjected to LPS-induced kidney injury.

**Fig 2:**
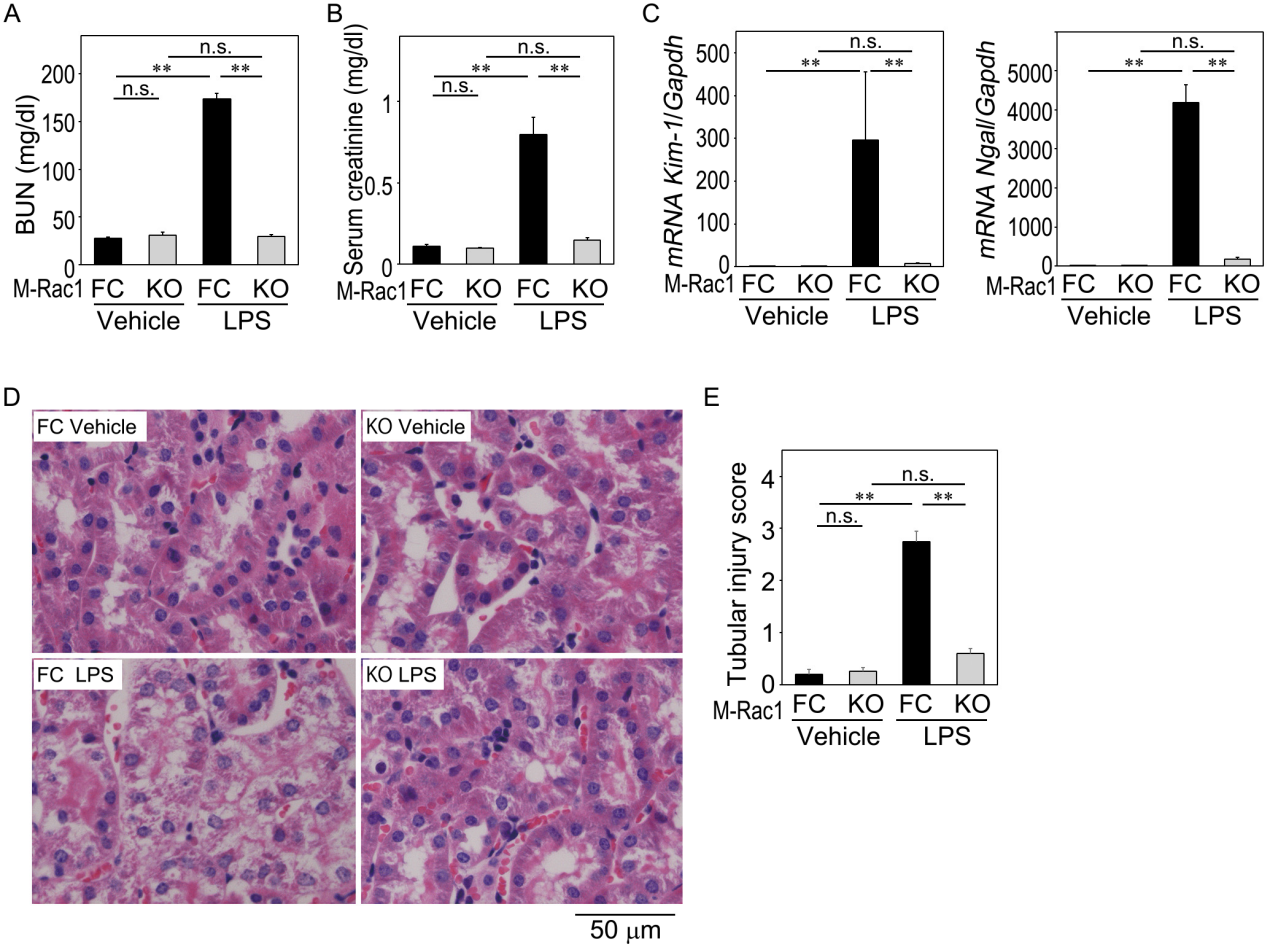
Lipopolysaccharide (LPS)-evoked renal functional and histological changes in M-Rac1 FC and KO mice. Vehicle or LPS (5 mg/kg) was injected intraperitoneally in M-Rac1 FC and KO mice, and animals were sacrificed 48 h later. (A and B) Blood urea nitrogen (BUN) and serum creatinine. Data are expressed as means ± s.e.m. Statistical analysis was performed by two-way ANOVA, *P* < 0.01 genotype effect, *P* < 0.01 treatment effect, *P* < 0.01 interaction effect. **P < 0.01 by Bonferroni's *post hoc* test. *n* = 8 per each group. (C) The mRNA expression of kidney injury biomarkers, *Kim-1* and *Ngal*, in the kidney homogenates of Vehicle- or LPS-injected M-Rac1 FC and KO mice. The mRNA levels were compared using real-time quantitative RT-PCR and expressed relative to M-Rac1 FC Vehicle group. Statistical analysis was performed by two-way ANOVA, *P* < 0.01 genotype effect, *P* < 0.01 treatment effect, *P* < 0.01 interaction effect. **P < 0.01 by Bonferroni's *post hoc* test. *n* = 8 per each group. (D) Typical images of hematoxylin-eosin stained kidney sections. Original magnification x 400. (E) Semiquantitative analysis of tubular injury. Data were analyzed using nonparametric analysis with Kruskal-Wallis test. **P < 0.01 by Mann-Whitney U test. *n* = 5 per each group.

At 48 h after LPS injection, BUN and serum creatinine were elevated in control M-Rac1 FC mice (Fig 2A and 2B). LPS-mediated elevations of BUN and serum creatinine were markedly attenuated in M-Rac1 KO mice. Histologically, LPS evoked tubular damage, such as loss of the apical brush border membrane and tubular vacuolization in M-Rac1 FC mice, which were significantly ameliorated in M-Rac1 KO mice (Fig 2D and 2E). Consistent with these observations, LPS enhanced mRNA expression of kidney injury biomarkers kidney injury molecule-1 (*Kim-1*) and neutrophil gelatinase-associated lipocalin (*Ngal*) in M-Rac1 FC mice, while myeloid Rac1 ablation prevented the LPS-invoked *Kim-1* and *Ngal* increment (Fig 2C). The survival rate up to 14 days after LPS injection did not significantly differ between M-Rac1 FC and KO mice (S1 Fig) Taken together, Rac1 deficiency in myeloid cells protected against renal functional and structural deterioration induced by LPS.

### Possible mechanisms of LPS-evoked renal injury and its amelioration by myeloid Rac1 deletion

**Fig 3:**
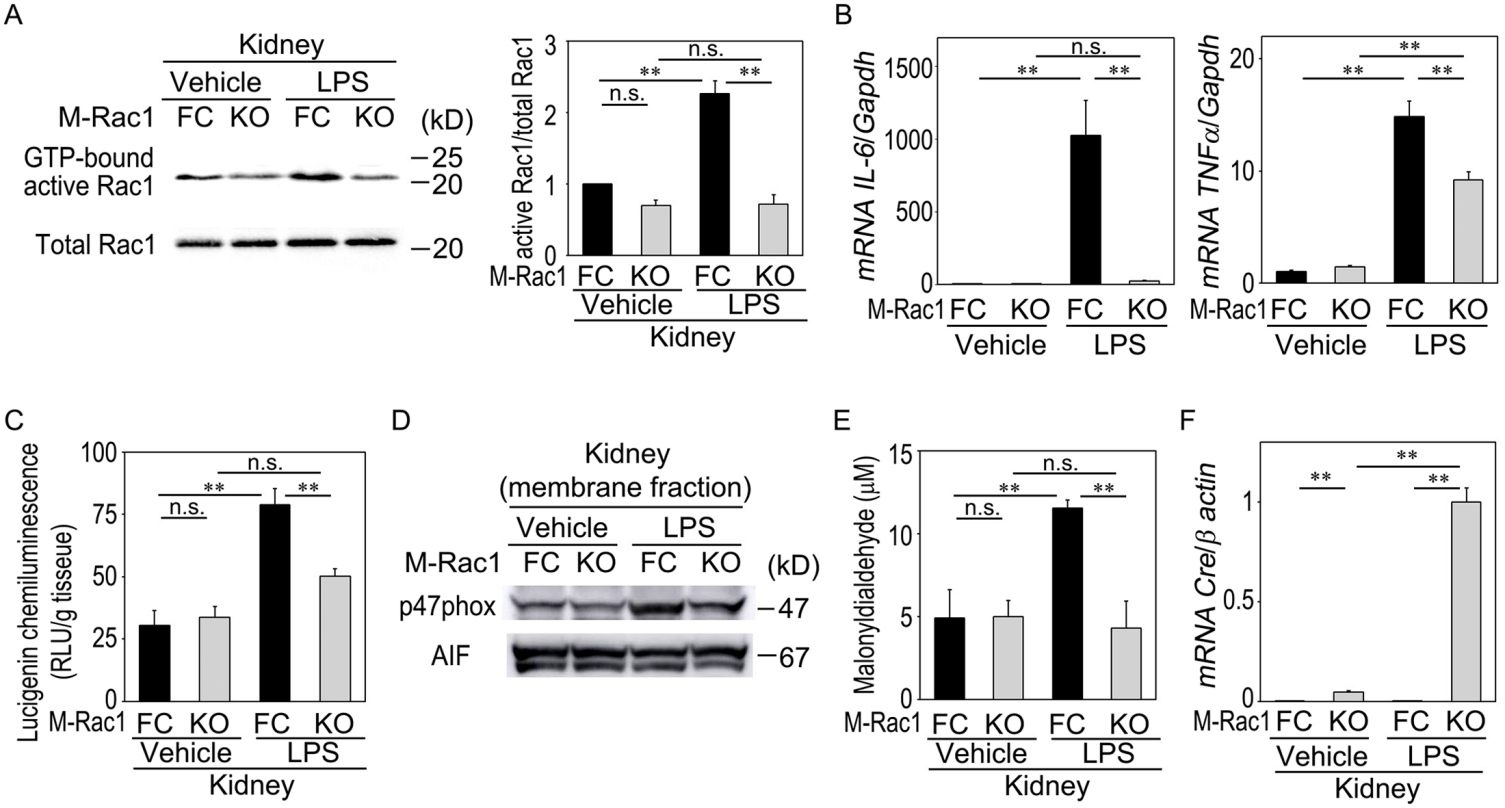
Effects of LPS injection on Rac1 activity, macrophage-related cytokine mRNA expression, NADPH oxidase activity, and ROS production in M-Rac1 FC and KO mice. (A) Expression of GTP-bound active Rac1 in the kidneys from Vehicle- or LPS-injected M-Rac1 FC and KO mice, as evaluated by GST pull-down assay. Left panel, representative blots. Right panel, result of densitometric analysis. Data are means ± s.e.m. Statistical analysis was performed by two-way ANOVA, *P* < 0.01 genotype effect, *P* < 0.01 treatment effect, *P* < 0.01 interaction effect. **P < 0.01 by Bonferroni's *post hoc* test. *n* = 3 per each group. (B) The mRNA levels of macrophage M1 cytokines, *IL-6* and *TNFα*, in the kidney homogenates. Statistical analysis was performed by two-way ANOVA, *P* < 0.01 genotype effect, *P* < 0.01 treatment effect, *P* < 0.01 interaction effect. **P < 0.01 by Bonferroni's *post hoc* test. *n* = 8 per each group. (C) NADPH oxidase activity in the kidney was expressed as ROS generation in the presence of NADPH by the lucigenin chemiluminescence method. Statistical analysis was performed by two-way ANOVA, *P* < 0.01 genotype effect, *P* < 0.01 treatment effect, *P* < 0.01 interaction effect. **P < 0.01 by Bonferroni's *post hoc* test. *n* = 5 per each group. (D) Representative immunoblot of p47phox in the membrane fraction of the kidney. AIF was used as a loading control. (E) ROS in the kidney homogenates were measured as malonyldialdehyde using TBARS Assay Kit. Statistical analysis was performed by two-way ANOVA, *P* < 0.01 genotype effect, *P* < 0.05 treatment effect, *P* < 0.01 interaction effect. **P < 0.01 by Bonferroni's *post hoc* test. *n* = 4 per each group. (F) Expression of *cre* mRNA in the kidneys from Vehicle- or LPS-injected M-Rac1 FC and KO mice. Statistical analysis was performed by two-way ANOVA, *P* < 0.01 genotype effect, *P* < 0.01 treatment effect, *P* < 0.01 interaction effect. **P < 0.01 by Bonferroni's *post hoc* test. *n* = 5 per each group.

We investigated the mechanisms by which M-Rac1 KO mice protected against LPS-mediated renal injury. First, we evaluated Rac1 activity in the kidneys of 4 groups of mice (Fig 3A). Basal Rac1 activity in the kidney did not differ between M-Rac1 FC and KO mice. LPS challenge increased GTP-bound active Rac1 in M-Rac1 FC mice, but did not activate Rac1 in M-Rac1 KO mice. These results suggest pivotal role for myeloid Rac1 as a trigger of LPS-induced kidney injury.

Macrophage-mediated inflammation has been implicated in the pathogenesis of tubulointerstitial disease.[2] Therefore, we examined macrophage-derived inflammatory cytokines. LPS strikingly increased *IL-6* mRNA expression in the kidney homogenates in M-Rac1 FC mice (1027-fold vs. Vehicle group, *P* < 0.01), while LPS-evoked *IL-6* induction was prominently suppressed in M-Rac1 KO mice (only 8-fold vs. Vehicle group) (Fig 3B). Gene expression profile of TNFα was similarly associated with the changes in renal function.

Rac1 is a component of NADPH oxidase complex (NOX2 isoform) in macrophages, and upon activation, Rac1 stimulates translocation of NADPH oxidase regulatory subunits p47phox and p67phox from cytosol to membrane, resulting in NADPH oxidase activation and ROS production.[23, 24] We found that NADPH oxidase activity in the kidney, measured as ROS generation in the presence of NADPH by the lucigenin chemiluminescence method, was significantly elevated in response to LPS injection in M-Rac1 FC mice (Fig 3C). NADPH oxidase activation was accompanied by the increased expression of p47phox in the membrane fraction (Fig 3D). ROS generation, as determined by TBARS assay, was also augmented in LPS-treated M-Rac1 FC mice (Fig 3E). These changes did not occur in M-Rac1 KO mice, in which LPS did not activate Rac1 in the kidney.

These data suggest the involvement of Rac1-NADPH oxidase-ROS-cytokine cascade in LPS-evoked renal tubular injury.

### Effects of LPS injection on macrophage and neutrophil accumulation in the kidney of M-Rac1 FC and KO mice

**Fig 4:**
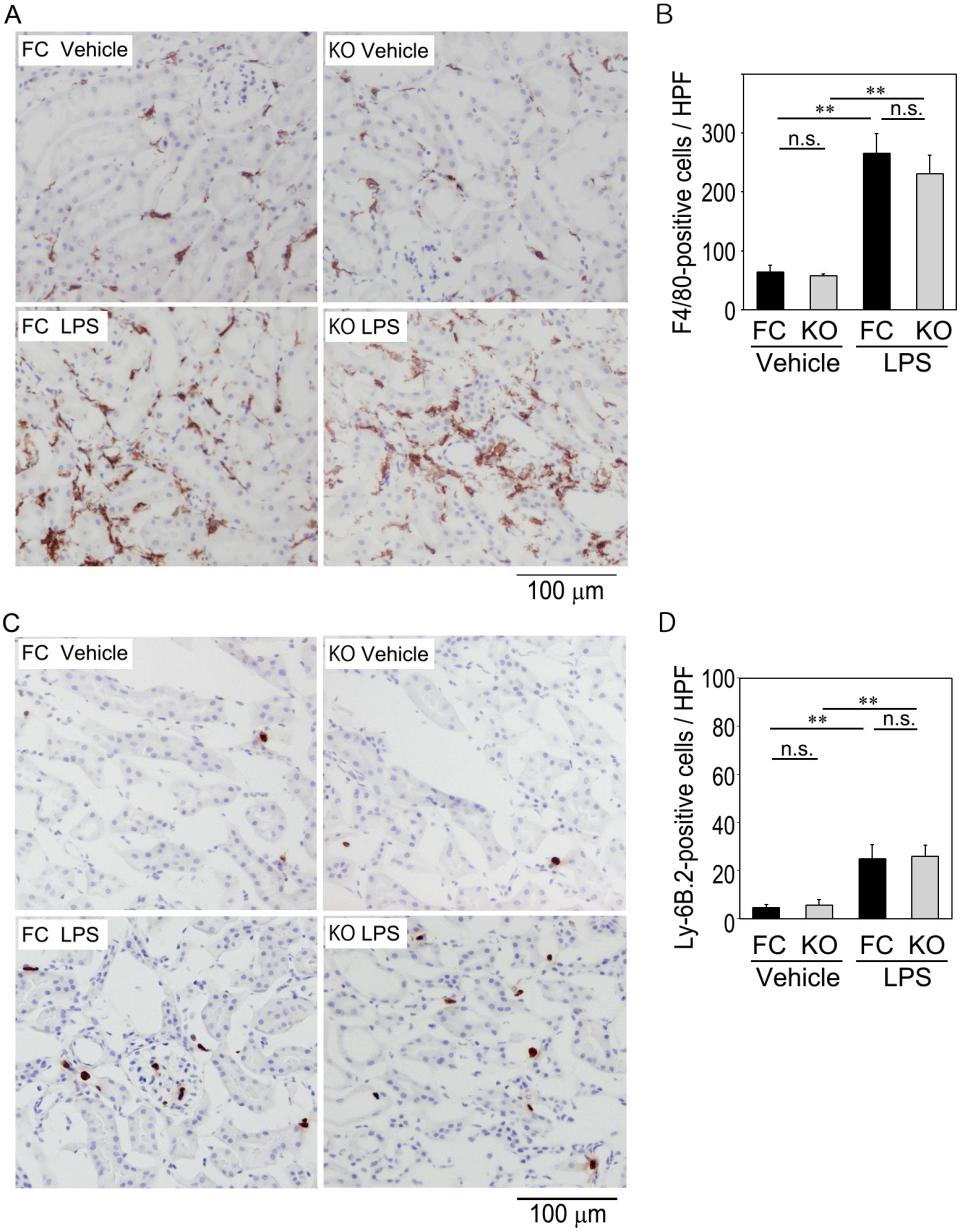
Immunostainings of macrophages and neutrophils in the kidneys of M-Rac1 FC and KO mice. (A) Representative micrographs of F4/80 immunostaining in the kidneys of Vehicle- or LPS-injected M-Rac1 FC and KO mice. Original magnification x 200. (B) Quantitative analysis of F4/80-positive macrophages. Data are means ± s.e.m. Statistical analysis was performed by two-way ANOVA, n.s. genotype effect, *P* < 0.01 treatment effect, n.s. interaction effect. **P < 0.01 by Bonferroni's *post hoc* test. *n* = 5 per each group. (C) Representative micrographs of Ly-6B.2 (mAb 7/4) immunostaining. (D) Quantitative analysis of Ly-6B.2-positive neutrophils. Statistical analysis was performed by two-way ANOVA, n.s. genotype effect, *P* < 0.01 treatment effect, n.s. interaction effect. **P < 0.01 by Bonferroni's *post hoc* test. *n* = 6 per group.

We next tested whether lack of cytokine induction in LPS-injected M-Rac1 KO mice might be attributed to the failure of myeloid cell infiltration into the kidney. LPS administration greatly elevated *cre* mRNA expression in the kidney of M-Rac1 KO mice (21-fold vs. Vehicle group, Fig 3F), suggesting that LPS accelerated the recruitment of cre-expressing macrophages and/or neutrophils to the kidney in M-Rac1 KO mice. Immunohistochemical analysis revealed that the number of macrophages in the kidney, as evaluated by F4/80 immunostaining, increased in response to LPS in M-Rac1 FC mice (Fig 4A and 4B). Similarly, LPS enhanced accumulation of F4/80-positive macrophages in M-Rac1 KO mice. As for neutrophils, much smaller numbers of Ly-6B.2 (mAb 7/4)-positive neutrophils were detected in the kidney. LPS slightly, but significantly, increased neutrophil accumulation, the extent of which did not differ between M-Rac1 FC and KO mice (Fig 4C and 4D). Collectively, improvement of LPS-induced renal injury in M-Rac1 KO mice was not related to the blockage of myeloid cell infiltration into the kidney, but associated with altered properties of macrophages with Rac1 inactivation and suppressed NADPH oxidase activity, ROS generation, and *IL-6* and *TNFα* induction in response to LPS.

### Involvement of macrophage Rac1 signaling in LPS-evoked cytokine production

Our *in vivo* study suggests critical role for myeloid Rac1, especially macrophage Rac1, in LPS-mediated renal inflammation and tubular injury. To obtain supportive evidence for the participation of macrophage Rac1, we performed *in vitro* studies using the cultured mouse macrophage cell line, RAW264.7, and explored the direct link between Rac1 signal cascade and LPS-triggered cytokine generation.

**Fig 5:**
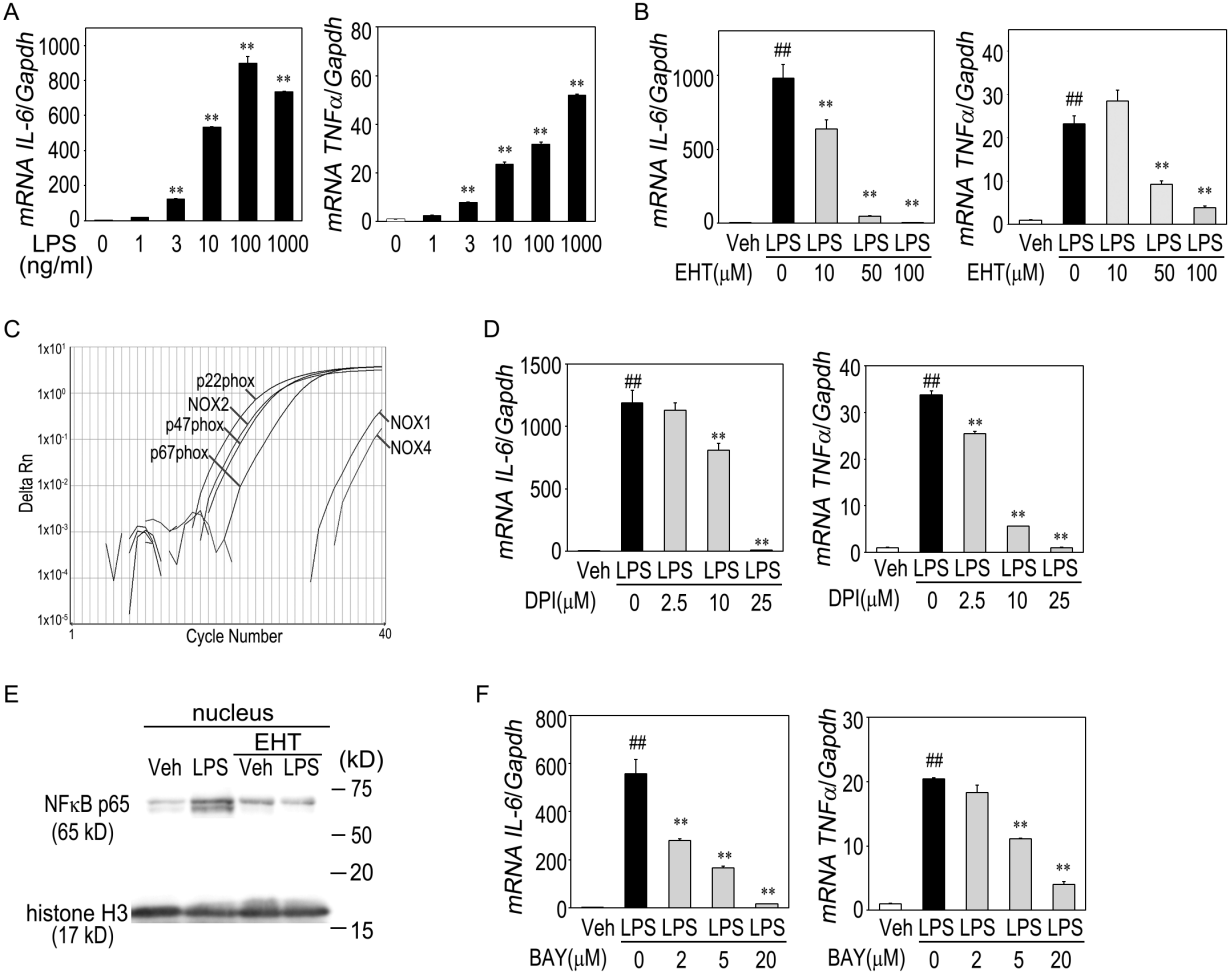
LPS-triggered cytokine mRNA induction and its underlying mechanisms in cultured macrophages. (A) Dose dependency of *IL-6* and *TNFα* mRNA induction in response to LPS (1, 3, 10, 100, and 1000 ng/ml) for 3 h in the cultured macrophage cell line RAW264.7. Data are means ± s.e.m. Statistical analysis was performed by one-way ANOVA. **P < 0.01 vs. Vehicle. *n* = 4 per each group. (B) Effects of pretreatment with the Rac1 inhibitor EHT1864 (10, 50, and 100 μM) on LPS (100 ng/ml)-evoked mRNA induction of *IL-6* and *TNFα*. Statistical analysis was performed by one-way ANOVA. ^##^*P* < 0.01 vs. Vehicle; **P < 0.01 vs. LPS without inhibitors. *n* = 4 per group. (C) The mRNA expression of NADPH oxidase components, NOX1, NOX2, NOX4, p22phox, p47phox, and p67phox, in cultured macrophage RAW264.7. (D) Effects of preincubation with the NADPH oxidase inhibitor diphenyleneiodonium (DPI: 2.5, 10, and 25 μM). Statistical analysis was performed by one-way ANOVA. ^##^*P* < 0.01 vs. Vehicle; **P < 0.01 vs. LPS without inhibitors. *n* = 4 per group. (E) Representative immunoblots of NF-κB p65 in the nuclear fraction. Histone H3 was used as a loading control. (F) Effects of preincubation with the NF-κB inhibitor BAY11-7082 (2, 5, and 20 μM). Statistical analysis was performed by one-way ANOVA. ^##^*P* < 0.01 vs. Vehicle; **P < 0.01 vs. LPS without inhibitors. *n* = 4 per group.

Exposure of cultured macrophages to LPS for 3 h dose-dependently enhanced *IL-6* and *TNFα* gene expression (Fig 5A). Consistent with the *in vivo* studies, the LPS (100 ng/ml)-mediated *IL-6* and *TNFα* mRNA induction was significantly attenuated by pretreatment with the Rac1 inhibitor EHT1864 (10, 50, and 100 μM) in a dose-dependent manner (Fig 5B). Among NADPH oxidase isoforms, NOX2 and its multicomplex components, p22phox, p47phox, and p67phox, were highly expressed, while expressions of NOX1 and NOX4 were low in this macrophage cell line (Fig 5C). The NADPH oxidase inhibitor DPI suppressed the LPS-induced *IL-6* and *TNFα* induction in a dose-dependent fashion (Fig 5D). Furthermore, LPS promoted the nuclear translocation of NF-κB p65, which was blocked by EHT1864 (Fig 5E). Indeed, the NF-kB inhibitor BAY11-7082 (2, 5, and 20 μM) dose dependently alleviated the LPS-evoked *IL-6* and *TNFα* mRNA upregulation (Fig 5F). These results indicated crucial role of Rac1 signaling pathway in the LPS-triggered *IL-6* and *TNFα* overproduction in macrophages.

The *in vivo* and *in vitro* studies corroborate our hypothesis that Rac1 in myeloid cells, especially macrophages, facilitates the LPS-evoked cytokine production via activation of NADPH oxidase, thereby accelerating inflammation-mediated kidney injury.

## Discussion

In the present study, we established the myeloid lineage-specific Rac1 KO mice, and investigated whether Rac1 in the myeloid cells contributes to inflammation-mediated kidney impairment. LPS injection in control mice resulted in elevation of BUN and serum creatinine, and tubular damage, which were associated with macrophage infiltration, activation of Rac1 and NADPH oxidase, and overproduction of ROS and macrophage-derived cytokines, *IL-6* and *TNFα*. Intriguingly, deletion of myeloid Rac1 protected against LPS-induced kidney injury. Renoprotection was accompanied by a failure of LPS to induce Rac1 activation, NADPH oxidase stimulation, enhance ROS and *IL-6* and *TNFα* mRNA expressions, although LPS promoted the accumulation of F4/80-positive macrophages. The *in vivo* findings were supported by *in vitro* experiments demonstrating that Rac1 inhibition prevented the LPS-evoked *IL-6* and *TNFα* induction in cultured macrophages. These findings suggest that Rac1 in the myeloid cells, especially macrophages, participates in the inflammatory mechanisms of kidney injury via cytokine overproduction.

Although the lysozyme M promoter directs the expression of all myeloid lineage cells including monocytes, macrophages, neutrophils, and some dendritic cells,[29] our results suggest that the deletion of Rac1 in macrophages constitutes a significant contribution to the KO phenotype for several reasons. Firstly, IL-6 and TNFα are major macrophage-derived M1 cytokines that convey macrophage-associated inflammation and organ injury. Their expressions in neutrophils are low compared to activated macrophages. Salkowski et al.[34] reported supportive evidence for macrophages as the primary producers of these cytokines, because liposome-mediated macrophage depletion resulted in >95% suppression of IL-6 induction and 50 to 75% reduction of TNFα mRNA in the liver of LPS-injected mice. Secondly, we found that the accumulation of Ly-6B.2 (mAb 7/4)-positive neutrophils was much less compared to F4/80-positive macrophages, which did not differ between M-Rac1 FC and KO mice. Thirdly, we showed that suppression of LPS-evoked IL-6 and TNFα overexpression by Rac1 blockade was reproduced in cultured macrophages. We further demonstrated that Rac1- NADPH oxidase-ROS-NF-κB pathway is involved in the LPS-mediated cytokine induction in the kidney *in vivo* and macrophages *in vitro*. Our *in vitro* data are consistent with an earlier study by Sanlioglu et al.,[35] showing that LPS-invoked Rac1 activation leads to TNFα production via NADPH oxidase-mediated ROS generation and resultant NF-κB stimulation in cultured macrophages. In their study, transfection of dominant negative Rac1 blocked ROS formation, nuclear translocation of NF-κB, and TNFα secretion following LPS challenge. The involvement of the Rac1-ROS-NF-κB-cytokine cascade in macrophages *in vivo* was suggested in hepatic ischemia/reperfusion injury, resulting in TNFα and inducible nitric oxide synthase gene induction.[36] Adenovirus-mediated inactivation of Rac1 actually suppressed the cascade of events elicited by ischemia/reperfusion. However, their study did not strictly distinguish the participation of Rac1 in macrophages from that in hepatocytes or other non-parenchymal cells.

Hematopoietic cells express both Rac1 and Rac2 isoforms. Rac1 is the predominant isoform in murine and human monocytes/macrophages.[37, 38] On the other hand, murine neutrophils have equivalent amounts of Rac1 and Rac2,[39] and Rac2 is the major isoform in human neutrophils.[40] Previously, several groups created Rac1 and Rac2 conditional KO mice specifically in hematopoietic cells. Wells et al. [37] established Rac1-deficient macrophages, using the type I interferon-inducible Mx1-Cre transgenic mice and Rac1^flox/flox^ mice. Their Rac1-deficient macrophages showed elongated morphology, diminished membrane ruffling, but normal migration and chemotaxis. Their results are compatible with our finding that LPS stimulated renal macrophage accumulation in M-Rac1 KO mice. In their study, NADPH oxidase activity was not evaluated. Glogauer et al.[41] generated conditional Rac1 deficiency restricted to cells of the granulocyte/monocyte lineage, using LysM-Cre transgenic mice and Rac1^flox/flox^ mice, similar to our mice. They analyzed neutrophil function *ex vivo*, by preparing neutrophil fraction from bone marrow cells. Their Rac1-deficient neutrophils exhibited defects in inflammatory recruitment, chemotaxis in response to fMLP, but normal superoxide production. In contrast, Rac2-null neutrophils showed defective superoxide production.[42] In most studies, macrophage or neutrophil functions were analyzed *ex vivo* by collecting specific fractions of cells from the mouse bone marrow. Their roles *in vivo* were not analyzed in detail.

Rac1 is a multi-functional molecule, acting as a key regulator of actin cytoskeleton, ROS generation, cell motility and gene transcription.[16] Rac1 has been implicated in a wide range of diseases, including cardiac hypertrophy, heart failure, arrhythmia,[22, 43] diabetic vascular complications,[44] Alzheimer disease,[45] pancreatitis and associated lung injury,[46] and metastatic cancers.[47] In the kidney, the role of podocyte Rac1 has garnered attention as etiology of foot process effacement, albuminuria, and glomerulosclerosis by affecting the cytoskeletal organization and cell motility.[16, 48] This is the first report demonstrating a pathogenic role of myeloid Rac1 as a mediator of inflammation and renal impairment, using the conditional KO mice. Macrophage infiltration and chronic inflammation are hallmarks of a variety of kidney diseases, including diabetic nephropathy, hypertensive nephrosclerosis, metabolic syndrome nephropathy, salt excess, renal ischemia/reperfusion injury, and obstructive nephropathy.[4, 49, 50] Rac1 in macrophages might be involved in the pathogenesis of these conditions.

Crosstalk of multiple cell types is implicated in the pathogenesis of kidney diseases. In the case of LPS-induced tubular injury, the LPS receptor, TLR4/MD2 complex, is expressed in vascular endothelial cells, tubular epithelial cells, glomerular cells, dendritic cells, macrophages, and B cells. Upon LPS challenge, LPS binds to TLR4 in vascular endothelial cells and tubular epithelial cells, and produce chemokines that stimulate recruitment of neutrophils and monocytes/macrophages in the interstitium around tubular epithelial cells. Subsequently, LPS binding to TLR4 in macrophages stimulates intracellular signal cascades leading to increased cytokines and interferon production via NF-kB and IRF3 activation. LPS finally causes tubular injury by acting directly on tubular cells or indirectly via macrophage-derived cytokines.[51] In the present study, we demonstrated that myeloid Rac1 deletion suppressed IL-6 and TNFα mRNA induction in the kidney, but did not reduce the numbers of F4/80-positive macrophages and Ly-6B.2-positive neutrophils in response to LPS challenge. The preserved accumulation of inflammatory cells in the kidney suggests that the initial monocytes/macrophages chemoattractant signals by the kidney cells (vascular endothelial cells, tubular epithelial cells, dendritic cells) and migration ability of monocytes/macrophages were not affected by myeloid Rac1 knockout. On the other hand, mRNA induction of macrophage-related cytokines (IL-6 and TNFα) and the resultant tubular injury were dependent on myeloid Rac1 pathway.

Unsuppressed infiltration of macrophages and neutrophils in the LPS-injected M-Rac1 KO mice might be caused by macrophage and neutrophil chemokines, monocyte chemoattractant protein-1 (MCP-1, also called Ccl2), Cxcl1, and Cxcl2. MCP-1 is a major monocyte/macrophage chemoattractant, while Cxcl1 and Cxcl2 enhance neutrophil recruitment. These chemokines are produced by tubular epithelial cells, vascular endothelial and smooth muscle cells, fibroblasts, neutrophils, monocytes, and macrophages. We quantitatively analyzed the mRNA expression profiles of these chemokines in the kidneys of Vehicle- and LPS-injected M-Rac1 FC and KO mice (S2 Fig). The MCP-1 mRNA expression was not significantly different between LPS-treated M-Rac1 FC and KO mice, which might contribute, at least in part, to unaltered macrophage infiltration in the LPS-injected KO mice. On the other hand, mRNA expressions of Cxcl1 and Cxcl2 were not correlated with the numbers of accumulated neutrophils; the expressions were markedly upregulated in the LPS-injected M-Rac1 FC mice, but attenuated in KO mice. Other factors, such as IL-8, might be involved in the infiltration of neutrophils in the KO group. In addition, further study is necessary to identify cellular sources of these chemokines.

In contrast to the myeloid-selective Rac1 KO mice, we previously reported that systemic inhibition of Rac1 by drug treatment blocked not only cytokine overexpression but also macrophage infiltration and MCP-1 mRNA induction in the kidney of obese diabetic mice, and salt-loaded angiotensin II-overproducing mice.[27, 28] Systemic administration of Rac1 inhibitor EHT1864 or NSC23766 inactivates Rac1 in all cell types. Several studies indicated that Rac1 in vascular endothelial cells may contribute to the macrophage infiltration via endothelial activation and MCP-1 induction.[52, 53] Together with our present findings, initial macrophage infiltration may be caused by MCP-1 produced by cells other than macrophages, such as vascular endothelial cells.

In the systemic Rac1 inhibition models, it is difficult to evaluate the influence of Rac1 inhibition on cytokine production within macrophages, because Rac1 inhibitor interrupts macrophage infiltration itself. On the other hand, macrophage accumulation occurred in myeloid cell-selective Rac1 KO mice subjected to LPS. In this sense, our model has an advantage in enabling analysis of the intracellular events within macrophages. We successfully identified the intracellular crosstalk between Rac1-NADPH oxidase-ROS-NF-κB pathway and LPS-triggered cytokine production cascade in macrophages.

In conclusion, M-Rac1 KO mice were resistant to LPS-evoked kidney dysfunction and tubular damage. Renoprotection correlated with suppression of Rac1 activation, NADPH oxidase activity, oxidative stress, and macrophage proinflammatory cytokines, IL-6 and TNFα, while M-Rac1 deletion did not block macrophage recruitment to the kidney. Indeed, we demonstrated that Rac1 signaling has crucial role in mediating LPS-evoked *IL-6* and *TNFα* mRNA upregulation via NADPH oxidase and NF-κB activation using cultured macrophage cell line. Taken these findings together, our data suggest that Rac1 in myeloid cells, especially macrophages, plays an important role in LPS-mediated cytokine overproduction, inflammation, and kidney injury. Rac1 in myeloid cells has potential as a novel target for the treatment of kidney disease.

## Supporting Information

S1 Fig Survival Analysis after LPS Injection in M-Rac1 FC and KO Mice. Survival rate was analyzed by Kaplan Meier method after LPS injection up to 14 days in M-Rac1 FC and KO mice (n = 10 for each group). Statistical analysis was performed by log rank test. n.s., not significant.
(TIF)

S2 Fig The mRNA Expression of Macrophage and Neutrophil Chemokines, *MCP-1*, *Cxcl1*, and *Cxcl2*, in the Kidney Homogenates of Vehicle- and LPS-Injected M-Rac1 FC and KO Mice. The mRNA levels were compared using real-time quantitative RT-PCR and expressed relative to M-Rac1 FC Vehicle group. Statistical analysis was performed by two-way ANOVA, *P* < 0.01 genotype effect, *P* < 0.01 treatment effect, *P* < 0.01 interaction effect. **P < 0.01 by Bonferroni's *post hoc* test. n.s., not significant. *n* = 8 per each group.
(TIF)

S3 Fig Original Uncropped Blots with Size Markers. (A) Original blots of Fig 1A. (B) Original blots of Fig 3A. (C) Original blots of Fig 3D. (D) Original blots of Fig 5E.
(TIF)

S1 Table Baseline Renal Function in M-Rac1 FC and KO Mice. Renal function was measured by collecting serum and urine from M-Rac1 FC and KO mice at 4 months of age. Data are means was.e.m (n = 6 for each group). Statistical analysis was performed by Student’s unpaired t-test. n.s., not significant.
(TIF)

## References

[1] Wang Y, Harris DC. Macrophages in renal disease. J Am Soc Nephrol. 2011;22(1):21–7. Epub 2011/01/07. 10.1681/asn.2010030269 . 21209251

[2] Sean Eardley K, Cockwell P. Macrophages and progressive tubulointerstitial disease. Kidney Int. 2005;68(2):437–55. Epub 2005/07/15. 10.1111/j.1523-1755.2005.00422.x . 16014021

[3] Muller DN, Kvakan H, Luft FC. Immune-related effects in hypertension and target-organ damage. Curr Opin Nephrol Hypertens. 2011;20(2):113–7. Epub 2011/01/20. 10.1097/MNH.0b013e3283436f88 . 21245763

[4] Franco M, Tapia E, Bautista R, Pacheco U, Santamaria J, Quiroz Y, et al Impaired pressure natriuresis resulting in salt-sensitive hypertension is caused by tubulointerstitial immune cell infiltration in the kidney. Am J Physiol Renal Physiol. 2013;304(7):F982–90. Epub 2013/02/01. 10.1152/ajprenal.00463.2012 . Pubmed Central PMCID: PMC3625854. 23364804 PMC3625854

[5] Ricardo SD, van Goor H, Eddy AA. Macrophage diversity in renal injury and repair. J Clin Invest. 2008;118(11):3522–30. Epub 2008/11/05. 10.1172/jci36150 . Pubmed Central PMCID: PMC2575702. 18982158 PMC2575702

[6] Sica A, Mantovani A. Macrophage plasticity and polarization: in vivo veritas. J Clin Invest. 2012;122(3):787–95. Epub 2012/03/02. 10.1172/jci59643 . Pubmed Central PMCID: PMC3287223. 22378047 PMC3287223

[7] Gomez Perdiguero E, Klapproth K, Schulz C, Busch K, Azzoni E, Crozet L, et al Tissue-resident macrophages originate from yolk-sac-derived erythro-myeloid progenitors. Nature. 2015;518(7540):547–51. Epub 2014/12/04. 10.1038/nature13989 . 25470051 PMC5997177

[8] Holdsworth SR, Neale TJ, Wilson CB. Abrogation of macrophage-dependent injury in experimental glomerulonephritis in the rabbit. Use of an antimacrophage serum. J Clin Invest. 1981;68(3):686–98. Epub 1981/09/01. . Pubmed Central PMCID: PMC370850. 7276168 10.1172/JCI110304 PMC370850

[9] Kitamoto K, Machida Y, Uchida J, Izumi Y, Shiota M, Nakao T, et al Effects of liposome clodronate on renal leukocyte populations and renal fibrosis in murine obstructive nephropathy. J Pharmacol Sci. 2009;111(3):285–92. Epub 2009/11/07. . 19893275 10.1254/jphs.09227fp

[10] Tomiyama-Hanayama M, Rakugi H, Kohara M, Mima T, Adachi Y, Ohishi M, et al Effect of interleukin-6 receptor blockage on renal injury in apolipoprotein E-deficient mice. Am J Physiol Renal Physiol. 2009;297(3):F679–84. Epub 2009/07/03. 10.1152/ajprenal.90680.2008 . 19570877

[11] Perez de Lema G, Maier H, Franz TJ, Escribese M, Chilla S, Segerer S, et al Chemokine receptor Ccr2 deficiency reduces renal disease and prolongs survival in MRL/lpr lupus-prone mice. J Am Soc Nephrol. 2005;16(12):3592–601. Epub 2005/11/04. 10.1681/asn.2005040426 . 16267157

[12] Wang Y, Wang YP, Zheng G, Lee VW, Ouyang L, Chang DH, et al Ex vivo programmed macrophages ameliorate experimental chronic inflammatory renal disease. Kidney Int. 2007;72(3):290–9. Epub 2007/04/19. 10.1038/sj.ki.5002275 . 17440493

[13] Cao Q, Wang Y, Zheng D, Sun Y, Wang Y, Lee VW, et al IL-10/TGF-beta-modified macrophages induce regulatory T cells and protect against adriamycin nephrosis. J Am Soc Nephrol. 2010;21(6):933–42. Epub 2010/03/20. 10.1681/asn.2009060592 . Pubmed Central PMCID: PMC2900959. 20299353 PMC2900959

[14] Ridley AJ, Paterson HF, Johnston CL, Diekmann D, Hall A. The small GTP-binding protein rac regulates growth factor-induced membrane ruffling. Cell. 1992;70(3):401–10. Epub 1992/08/07. . 1643658 10.1016/0092-8674(92)90164-8

[15] Abo A, Pick E, Hall A, Totty N, Teahan CG, Segal AW. Activation of the NADPH oxidase involves the small GTP-binding protein p21rac1. Nature. 1991;353(6345):668–70. Epub 1991/10/17. 10.1038/353668a0 . 1922386

[16] Nagase M, Fujita T. Role of Rac1-mineralocorticoid-receptor signalling in renal and cardiac disease. Nat Rev Nephrol. 2013;9(2):86–98. Epub 2013/01/09. 10.1038/nrneph.2012.282 . 23296296

[17] Sugihara K, Nakatsuji N, Nakamura K, Nakao K, Hashimoto R, Otani H, et al Rac1 is required for the formation of three germ layers during gastrulation. Oncogene. 1998;17(26):3427–33. Epub 1999/02/25. 10.1038/sj.onc.1202595 . 10030666

[18] Benitah SA, Frye M, Glogauer M, Watt FM. Stem cell depletion through epidermal deletion of Rac1. Science. 2005;309(5736):933–5. Epub 2005/08/06. 10.1126/science.1113579 . 16081735

[19] Kassai H, Terashima T, Fukaya M, Nakao K, Sakahara M, Watanabe M, et al Rac1 in cortical projection neurons is selectively required for midline crossing of commissural axonal formation. Eur J Neurosci. 2008;28(2):257–67. Epub 2008/08/16. 10.1111/j.1460-9568.2008.06343.x . 18702697

[20] Tan W, Palmby TR, Gavard J, Amornphimoltham P, Zheng Y, Gutkind JS. An essential role for Rac1 in endothelial cell function and vascular development. FASEB J. 2008;22(6):1829–38. Epub 2008/02/05. 10.1096/fj.07-096438 . 18245172

[21] Sawada N, Kim HH, Moskowitz MA, Liao JK. Rac1 is a critical mediator of endothelium-derived neurotrophic activity. Sci Signal. 2009;2(61):ra10 Epub 2009/03/13. 10.1126/scisignal.2000162 . Pubmed Central PMCID: PMC2668716. 19278959 PMC2668716

[22] Satoh M, Ogita H, Takeshita K, Mukai Y, Kwiatkowski DJ, Liao JK. Requirement of Rac1 in the development of cardiac hypertrophy. Proc Natl Acad Sci U S A. 2006;103(19):7432–7. Epub 2006/05/03. 10.1073/pnas.0510444103 . Pubmed Central PMCID: PMC1455410. 16651530 PMC1455410

[23] Lambeth JD. NOX enzymes and the biology of reactive oxygen. Nat Rev Immunol. 2004;4(3):181–9. Epub 2004/03/25. 10.1038/nri1312 . 15039755

[24] Sedeek M, Nasrallah R, Touyz RM, Hebert RL. NADPH oxidases, reactive oxygen species, and the kidney: friend and foe. J Am Soc Nephrol. 2013;24(10):1512–8. Epub 2013/08/24. 10.1681/asn.2012111112 . Pubmed Central PMCID: PMC3785272. 23970124 PMC3785272

[25] Shibata S, Nagase M, Yoshida S, Kawarazaki W, Kurihara H, Tanaka H, et al Modification of mineralocorticoid receptor function by Rac1 GTPase: implication in proteinuric kidney disease. Nat Med. 2008;14(12):1370–6. Epub 2008/11/26. 10.1038/nm.1879 . 19029984

[26] Shibata S, Mu S, Kawarazaki H, Muraoka K, Ishizawa K, Yoshida S, et al Rac1 GTPase in rodent kidneys is essential for salt-sensitive hypertension via a mineralocorticoid receptor-dependent pathway. J Clin Invest. 2011;121(8):3233–43. Epub 2011/07/19. 10.1172/jci43124 . Pubmed Central PMCID: PMC3148723. 21765214 PMC3148723

[27] Kawarazaki W, Nagase M, Yoshida S, Takeuchi M, Ishizawa K, Ayuzawa N, et al Angiotensin II- and salt-induced kidney injury through Rac1-mediated mineralocorticoid receptor activation. J Am Soc Nephrol. 2012;23(6):997–1007. Epub 2012/03/24. 10.1681/asn.2011070734 . Pubmed Central PMCID: PMC3358757. 22440899 PMC3358757

[28] Yoshida S, Ishizawa K, Ayuzawa N, Ueda K, Takeuchi M, Kawarazaki W, et al Local mineralocorticoid receptor activation and the role of Rac1 in obesity-related diabetic kidney disease. Nephron Exp Nephrol. 2014;126(1):16–24. Epub 2014/03/08. 10.1159/000358758 . 24603367

[29] Clausen BE, Burkhardt C, Reith W, Renkawitz R, Forster I. Conditional gene targeting in macrophages and granulocytes using LysMcre mice. Transgenic Res. 1999;8(4):265–77. Epub 2000/01/06. . 10621974 10.1023/a:1008942828960

[30] Rickard AJ, Morgan J, Tesch G, Funder JW, Fuller PJ, Young MJ. Deletion of mineralocorticoid receptors from macrophages protects against deoxycorticosterone/salt-induced cardiac fibrosis and increased blood pressure. Hypertension. 2009;54(3):537–43. Epub 2009/07/29. 10.1161/hypertensionaha.109.131110 . 19635989

[31] Shutes A, Onesto C, Picard V, Leblond B, Schweighoffer F, Der CJ. Specificity and mechanism of action of EHT 1864, a novel small molecule inhibitor of Rac family small GTPases. J Biol Chem. 2007;282(49):35666–78. Epub 2007/10/13. 10.1074/jbc.M703571200 . 17932039

[32] Hamers AA, van Dam L, Teixeira Duarte JM, Vos M, Marinkovic G, van Tiel CM, et al Deficiency of Nuclear Receptor Nur77 Aggravates Mouse Experimental Colitis by Increased NFkappaB Activity in Macrophages. PLoS One. 2015;10(8):e0133598 Epub 2015/08/05. 10.1371/journal.pone.0133598 . Pubmed Central PMCID: PMC4524678. 26241646 PMC4524678

[33] Shibata S, Nagase M, Yoshida S, Kawachi H, Fujita T. Podocyte as the target for aldosterone: roles of oxidative stress and Sgk1. Hypertension. 2007;49(2):355–64. Epub 2007/01/04. 10.1161/01.HYP.0000255636.11931.a2 . 17200434

[34] Salkowski CA, Neta R, Wynn TA, Strassmann G, van Rooijen N, Vogel SN. Effect of liposome-mediated macrophage depletion on LPS-induced cytokine gene expression and radioprotection. J Immunol. 1995;155(6):3168–79. Epub 1995/09/15. . 7673730

[35] Sanlioglu S, Williams CM, Samavati L, Butler NS, Wang G, McCray PB Jr., et al Lipopolysaccharide induces Rac1-dependent reactive oxygen species formation and coordinates tumor necrosis factor-alpha secretion through IKK regulation of NF-kappa B. J Biol Chem. 2001;276(32):30188–98. Epub 2001/06/13. 10.1074/jbc.M102061200 . 11402028

[36] Harada N, Iimuro Y, Nitta T, Yoshida M, Uchinami H, Nishio T, et al Inactivation of the small GTPase Rac1 protects the liver from ischemia/reperfusion injury in the rat. Surgery. 2003;134(3):480–91. Epub 2003/10/14. . 14555937 10.1067/s0039-6060(03)00256-3

[37] Wells CM, Walmsley M, Ooi S, Tybulewicz V, Ridley AJ. Rac1-deficient macrophages exhibit defects in cell spreading and membrane ruffling but not migration. J Cell Sci. 2004;117(Pt 7):1259–68. Epub 2004/03/05. 10.1242/jcs.00997 . 14996945

[38] Zhao X, Carnevale KA, Cathcart MK. Human monocytes use Rac1, not Rac2, in the NADPH oxidase complex. J Biol Chem. 2003;278(42):40788–92. Epub 2003/08/13. 10.1074/jbc.M302208200 . 12912997

[39] Li S, Yamauchi A, Marchal CC, Molitoris JK, Quilliam LA, Dinauer MC. Chemoattractant-stimulated Rac activation in wild-type and Rac2-deficient murine neutrophils: preferential activation of Rac2 and Rac2 gene dosage effect on neutrophil functions. J Immunol. 2002;169(9):5043–51. Epub 2002/10/23. . 12391220 10.4049/jimmunol.169.9.5043

[40] Heyworth PG, Bohl BP, Bokoch GM, Curnutte JT. Rac translocates independently of the neutrophil NADPH oxidase components p47phox and p67phox. Evidence for its interaction with flavocytochrome b558. J Biol Chem. 1994;269(49):30749–52. Epub 1994/12/09. . 7982999

[41] Glogauer M, Marchal CC, Zhu F, Worku A, Clausen BE, Foerster I, et al Rac1 deletion in mouse neutrophils has selective effects on neutrophil functions. J Immunol. 2003;170(11):5652–7. Epub 2003/05/22. . 12759446 10.4049/jimmunol.170.11.5652

[42] Gu Y, Filippi MD, Cancelas JA, Siefring JE, Williams EP, Jasti AC, et al Hematopoietic cell regulation by Rac1 and Rac2 guanosine triphosphatases. Science. 2003;302(5644):445–9. Epub 2003/10/18. 10.1126/science.1088485 . 14564009

[43] Brown JH, Del Re DP, Sussman MA. The Rac and Rho hall of fame: a decade of hypertrophic signaling hits. Circ Res. 2006;98(6):730–42. Epub 2006/04/01. 10.1161/01.RES.0000216039.75913.9e . 16574914

[44] Vecchione C, Aretini A, Marino G, Bettarini U, Poulet R, Maffei A, et al Selective Rac-1 inhibition protects from diabetes-induced vascular injury. Circ Res. 2006;98(2):218–25. Epub 2005/12/17. 10.1161/01.res.0000200440.18768.30 . 16357302

[45] Desire L, Bourdin J, Loiseau N, Peillon H, Picard V, De Oliveira C, et al RAC1 inhibition targets amyloid precursor protein processing by gamma-secretase and decreases Abeta production in vitro and in vivo. J Biol Chem. 2005;280(45):37516–25. Epub 2005/09/10. 10.1074/jbc.M507913200 . 16150730

[46] Binker MG, Binker-Cosen AA, Gaisano HY, Cosen-Binker LI. Inhibition of Rac1 decreases the severity of pancreatitis and pancreatitis-associated lung injury in mice. Exp Physiol. 2008;93(10):1091–103. Epub 2008/06/24. 10.1113/expphysiol.2008.043141 . 18567599

[47] Kawazu M, Ueno T, Kontani K, Ogita Y, Ando M, Fukumura K, et al Transforming mutations of RAC guanosine triphosphatases in human cancers. Proc Natl Acad Sci U S A. 2013;110(8):3029–34. Epub 2013/02/06. 10.1073/pnas.1216141110 . Pubmed Central PMCID: PMC3581941. 23382236 PMC3581941

[48] Mundel P, Reiser J. Proteinuria: an enzymatic disease of the podocyte? Kidney Int. 2010;77(7):571–80. Epub 2009/11/20. 10.1038/ki.2009.424 . Pubmed Central PMCID: PMC4109304. 19924101 PMC4109304

[49] Chow FY, Nikolic-Paterson DJ, Ozols E, Atkins RC, Rollin BJ, Tesch GH. Monocyte chemoattractant protein-1 promotes the development of diabetic renal injury in streptozotocin-treated mice. Kidney Int. 2006;69(1):73–80. Epub 2005/12/24. 10.1038/sj.ki.5000014 . 16374426

[50] Nagase M, Matsui H, Shibata S, Gotoda T, Fujita T. Salt-induced nephropathy in obese spontaneously hypertensive rats via paradoxical activation of the mineralocorticoid receptor: role of oxidative stress. Hypertension. 2007;50(5):877–83. Epub 2007/09/19. 10.1161/hypertensionaha.107.091058 . 17875821

[51] Wang Y, Wang DH. Role of the transient receptor potential vanilloid type 1 channel in renal inflammation induced by lipopolysaccharide in mice. Am J Physiol Regul Integr Comp Physiol. 2013;304(1):R1–9. Epub 2012/11/16. 10.1152/ajpregu.00163.2012 . Pubmed Central PMCID: PMC3543652. 23152109 PMC3543652

[52] Marinkovic G, Hibender S, Hoogenboezem M, van Broekhoven A, Girigorie AF, Bleeker N, et al Immunosuppressive drug azathioprine reduces aneurysm progression through inhibition of Rac1 and c-Jun-terminal-N-kinase in endothelial cells. Arterioscler Thromb Vasc Biol. 2013;33(10):2380–8. Epub 2013/08/21. 10.1161/atvbaha.113.301394 . 23950142

[53] Hwaiz R, Hasan Z, Rahman M, Zhang S, Palani K, Syk I, et al Rac1 signaling regulates sepsis-induced pathologic inflammation in the lung via attenuation of Mac-1 expression and CXC chemokine formation. J Surg Res. 2013;183(2):798–807. Epub 2013/04/03. 10.1016/j.jss.2013.02.045 . 23545410

