# Supplementary figures and images for: Deletion of Rac1GTPase in the Myeloid Lineage Protects against Inflammation-Mediated Kidney Injury in Mice
Source: PLoS One. 2016 Mar 3;11(3):e0150886. doi: 10.1371/journal.pone.0150886 (PMC4777421; doi:10.1371/journal.pone.0150886)

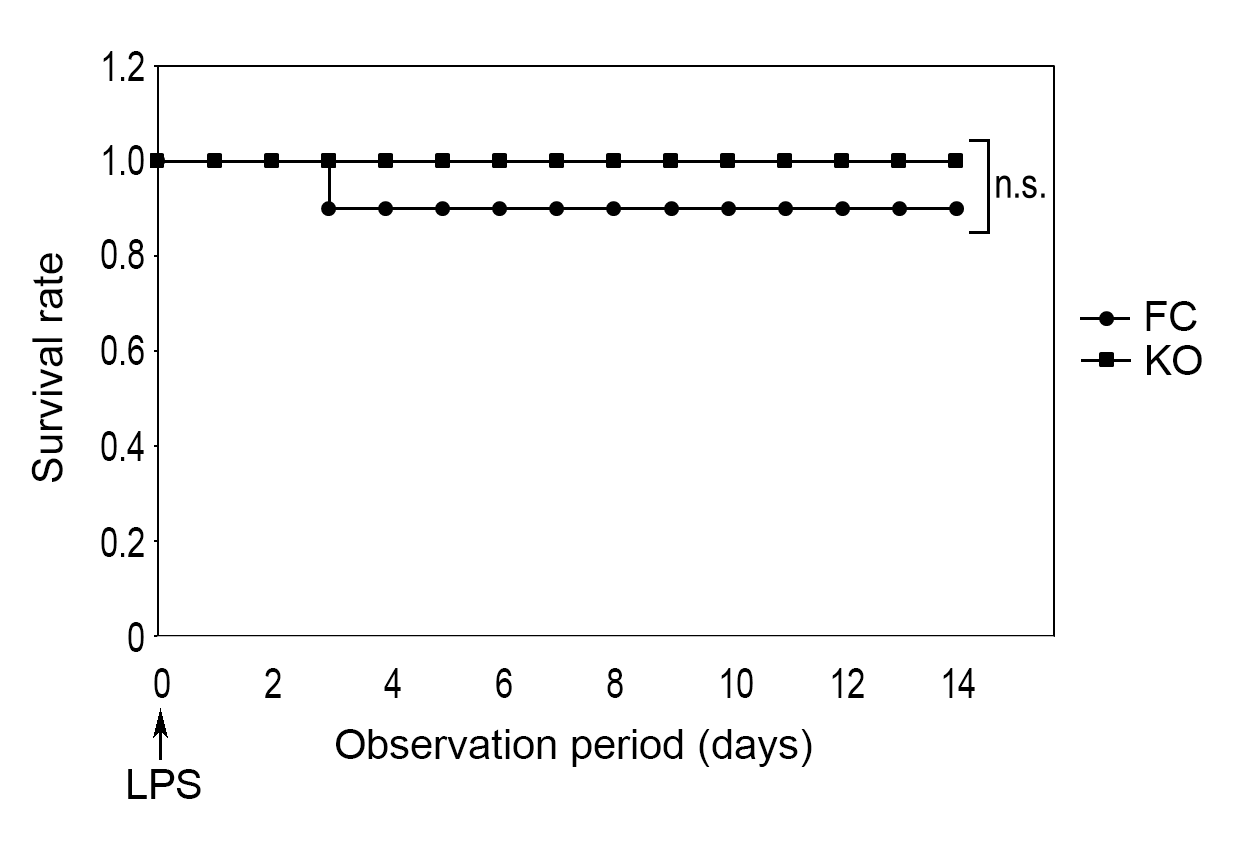

Supplement: S1 Fig — Survival rate was analyzed by Kaplan Meier method after LPS injection up to 14 days in M-Rac1 FC and KO mice (n = 10 for each group). Statistical analysis was performed by log rank test. n.s., not significant. (TIF) [file pone.0150886.s001.tif]

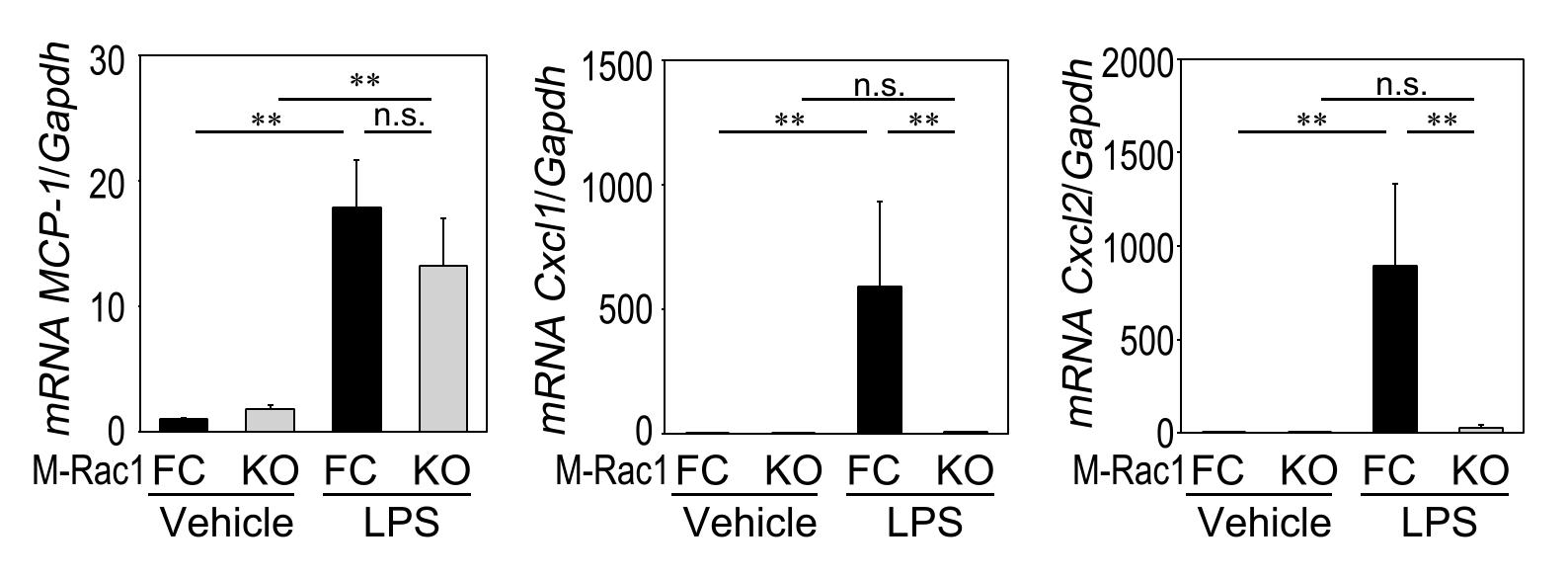

Supplement: S2 Fig — The mRNA levels were compared using real-time quantitative RT-PCR and expressed relative to M-Rac1 FC Vehicle group. Statistical analysis was performed by two-way ANOVA, P < 0.01 genotype effect, P < 0.01 treatment effect, P < 0.01 interaction effect. **P < 0.01 by Bonferroni's post hoc test. n.s., not significant. n = 8 per each group. (TIF) [file pone.0150886.s002.tif]

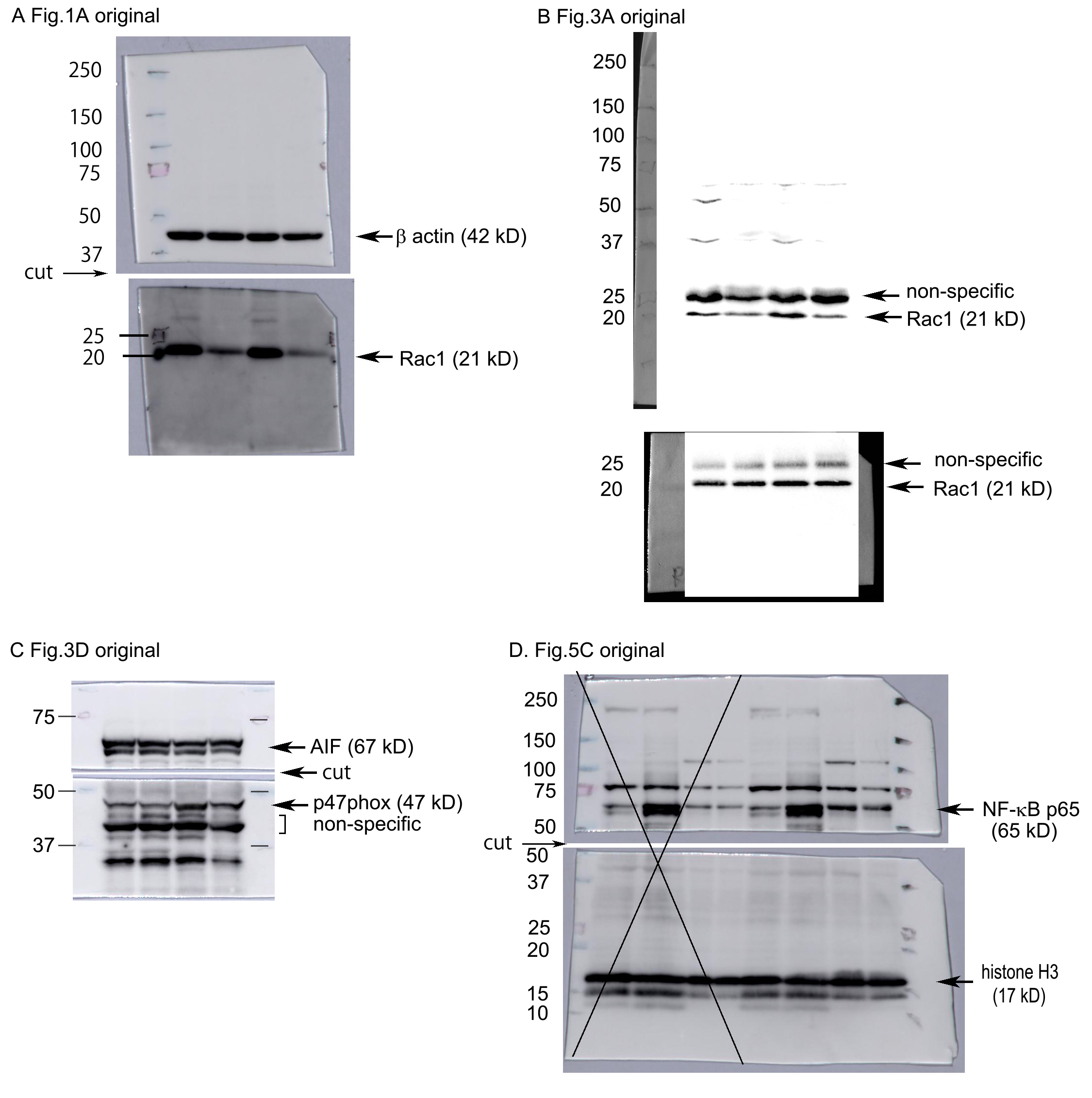

Supplement: S3 Fig — (A) Original blots of Fig 1A. (B) Original blots of Fig 3A. (C) Original blots of Fig 3D. (D) Original blots of Fig 5E. (TIF) [file pone.0150886.s003.tif]

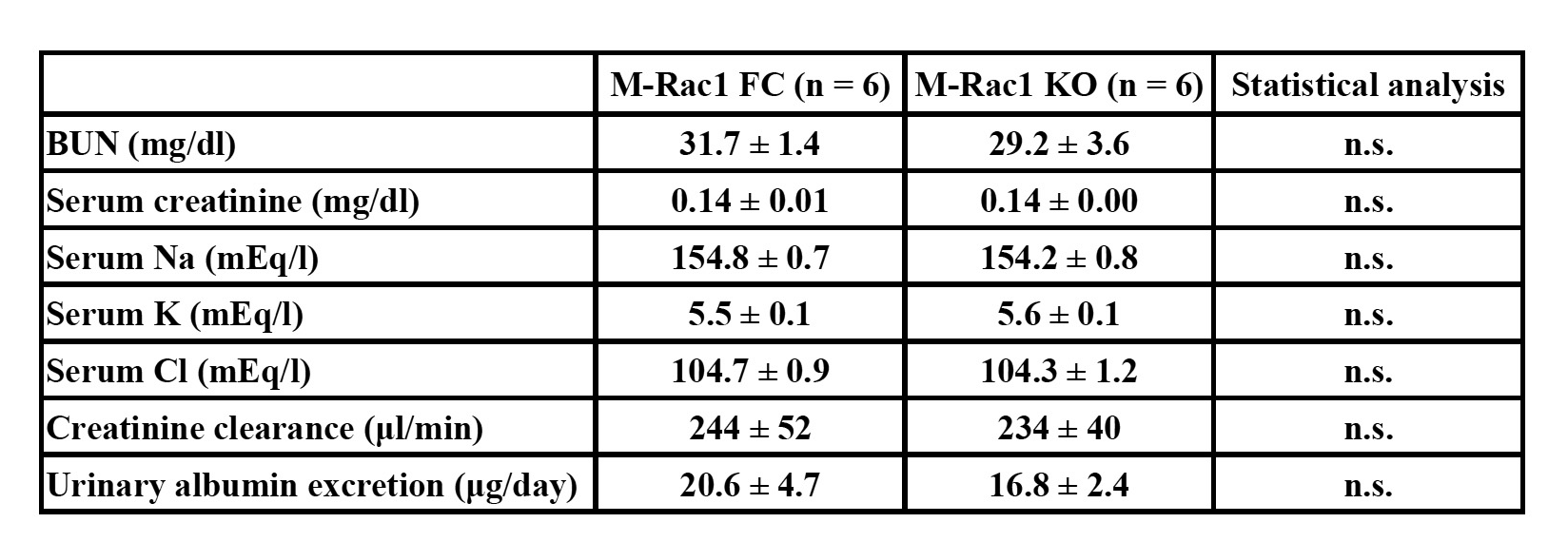

Supplement: S1 Table — Renal function was measured by collecting serum and urine from M-Rac1 FC and KO mice at 4 months of age. Data are means was.e.m (n = 6 for each group). Statistical analysis was performed by Student’s unpaired t-test. n.s., not significant. (TIF) [file pone.0150886.s004.tif]
